# Physical activity and the risk of cardiovascular disease, cirrhosis, cancer and mortality among individuals with MASLD: a prospective cohort study

**DOI:** 10.1136/bmjsem-2025-002702

**Published:** 2025-10-13

**Authors:** Sihua Xu, Yiyuan Xiao, Chaoyu Xu, Xiaoya Zeng, Liangyu Zhao, Tuojian Li, Deke Jiang, Rong Na, Haitao Chen

**Affiliations:** 1 Sun Yat-Sen University School of Public Health Shenzhen, Shenzhen, China; 2 Sun Yat-Sen University, Guangzhou, China; 3 Shandong University, Jinan, China; 4 Department of Infectious Diseases, Southern Medical University, Guangzhou, China; 5 Department of Surgery, The University of Hong Kong, Hong Kong, Hong Kong

**Keywords:** Physical activity, Accelerometer, Cardiovascular

## Abstract

**Objectives:**

Metabolic dysfunction-associated steatotic liver disease (MASLD) is a global health issue due to its high prevalence, yet the impact of accelerometer-measured physical activity on clinical outcomes remains unclear. This study aims to examine the associations of physical activity with the risk of liver cirrhosis, cancer, cardiovascular disease (CVD) incidence and mortality.

**Methods:**

32 681 MASLD participants with accelerometer-derived physical activity data from the UK Biobank were analysed. Physical activity intensity was categorised into light (LPA), moderate (MPA) and vigorous (VPA) intensity. Cox proportional hazard and acceleration failure models were employed to assess associations between physical activity duration and outcomes.

**Results:**

During a median follow-up of 7.5–7.9 years, 1883 deaths, 151 liver cirrhosis, 3312 cancers and 6657 CVD events were recorded. Physical activity, regardless of intensity, was consistently associated with a reduced risk of liver cirrhosis, CVD and all-cause mortality. Compared with non-MASLD individuals, our analysis indicates that longer duration of physical activity, specifically >1945 min/week of LPA or >383 min/week of MPA may theoretically eliminate the excess risk of mortality associated with MASLD.

**Conclusions:**

Among MASLD individuals, longer physical activity duration, regardless of intensity, was associated with reduced risks of liver cirrhosis and mortality. MPA and VPA were associated with lower CVD risk, while VPA was associated with reduced cancer risk, highlighting the potential benefits of increasing the intensity and duration of physical activity in MASLD management.

**WHAT IS ALREADY KNOWN ON THIS TOPIC**

- Metabolic dysfunction-associated steatotic liver disease (MASLD) is a prevalent condition with adverse health outcomes, but the role of physical activity in mitigating these risks is not well understood. Previous studies have highlighted the potential benefits of physical activity, but the impact of different intensities and durations of activity on clinical outcomes in patients remains unclear.

**WHAT THIS STUDY ADDS**

- This study demonstrates that longer durations of physical activity, regardless of intensity, are associated with reduced risks of liver cirrhosis and mortality. Moderate-intensity and vigorous-intensity physical activity durations were associated with lower cardiovascular disease risk, while vigorous-intensity physical activity duration was associated with reduced cancer risk.

**HOW THIS STUDY MIGHT AFFECT RESEARCH, PRACTICE OR POLICY**

- The findings underscore the importance of promoting physical activity, especially moderate- and vigorous-intensity activities, in MASLD management. This could inform future guidelines for physical activity interventions aimed at reducing the burden of MASLD and associated comorbidities, potentially influencing clinical practice and public health policies.

## Introduction

 Metabolic dysfunction-associated steatotic liver disease (MASLD), formerly known as non-alcoholic fatty liver disease (NAFLD), refers to diagnosed hepatic steatosis with at least one of the five cardiometabolic risk factors. The disease went through its second nomenclature process in June 2023, following the definition of metabolic dysfunction-associated fatty liver disease (MAFLD) in 2020.^1 2^ Over the past decades, the incidence of MASLD has shown a markedly rising trend due to the global increase of obesity and type 2 diabetes (T2D).^3^ Epidemiological evidence shows that MASLD affects one third of the global population.^4^

So far, there are no specific pharmacotherapies for MASLD, and it has become one of the major contributors to liver disease-related morbidity and mortality, posing a significant challenge to global public health.^5^,^7^ MASLD exhibits a wide range of clinical manifestations and symptoms, ranging from simple steatosis to liver fibrosis, cirrhosis and ultimately progressing to hepatocellular carcinoma (HCC).^8 9^ Liver-related complications, such as cirrhosis and HCC, are the leading causes of mortality among individuals with MASLD. Furthermore, MASLD forms a complex interaction network with metabolic conditions like obesity and T2D, significantly increasing the risk of mortality. Notably, the disease burden MASLD brings is not confined to liver-specific outcomes but also extrahepatic complications, including cardiovascular diseases (CVD), chronic kidney disease and cancers.^10^,^13^ Therefore, early intervention and risk management to prevent the progression of MASLD have become important public health concerns.

Physical activity, as a simple and effective lifestyle intervention, has been extensively studied and proven to confer metabolic benefits. Regular physical activity significantly reduces the risk of T2D, obesity and CVDs by improving insulin sensitivity, reducing visceral fat and modulating lipid metabolism.^14^,^16^ Furthermore, accumulating evidence suggests that physical activity plays a crucial role in lowering the risks of certain cancers and all-cause mortality, underscoring its importance in public health.^17^,^22^

Despite the well-documented benefits of physical activity in other metabolic conditions, its long-term impact on patients with MASLD remains unclear. Existing studies primarily focus on the short-term effects of physical activity on the hepatic biochemical markers of patients with MASLD, but its long-term influences on disease progression, such as liver fibrosis, cirrhosis, HCC and related mortality, were not clear.^23 24^ Another limitation of previous research is the reliance on self-reported physical activity data collected through questionnaires, which are prone to recall bias and insensitive to light-intensity physical activity (LPA), thus restricting the classification of physical activity into broad categories.^25^ In contrast, accelerometers, as wearable devices, can objectively measure the intensity and duration of physical activity over time under free-living conditions.^26 27^

In this study, using accelerometer-measured physical activity data of MASLD individuals, we prospectively examined the associations of physical activity with the risk of liver cirrhosis, cancer, CVD incidence and mortality, aiming to provide more targeted and effective physical activity recommendations for individuals with MASLD.

## Methods

### Study design and population

Data used in our study were obtained from the UK Biobank (UKB) (Application: 78559). The UKB is a large-scale prospective cohort study with over 500 000 participants aged 40–69 years when recruited in 2006–2010. The study has collected and continues to collect phenotypic and genotypic information from its participants, including data from questionnaires, physical measures, sample assays, accelerometry, multimodal imaging, genome-wide genotyping and longitudinal follow-up for a wide range of health-related outcomes.

188 192 individuals with MASLD from all UKB participants were enrolled in this study. We then excluded participants without sufficient information on accelerometry measurement-related data (n=153 095) and those with data of poor quality (n=2416). Ultimately, a total of 32 681 participants were retained. After the imputation of certain covariates, analyses were conducted using the imputed dataset. To examine the association of physical activity duration with specific outcomes, individuals were excluded if the outcome of interest (liver cirrhosis: n=58; cancer: n=4610 and CVD: n=10 747) occurred prior to the study baseline (the time of accelerometry data collection).

### Patient and public involvement statement

Patients or the public were not involved in the design, or conduct, or reporting or dissemination plans of our research.

### Diagnosis of MASLD

Considering the high consistency of three definitions,^28^,^31^ all individuals were included in the analysis, irrespective of whether they were diagnosed as NAFLD, MAFLD or MASLD. NAFLD was diagnosed using *International Classification of Diseases, Tenth Revision* code K75.8 and K76.0.^32^ MAFLD was diagnosed according to the 2020 consensus of an international panel of hepatologists.^2^ Participants were diagnosed with MAFLD if they exhibited hepatic steatosis defined by fatty liver index (the cut-off was ≥60) or the imaging data indicator, proton density fat fraction (PDFF, the cut-off was ≥5%) and reached any one of the three criteria: (1) overweight or obesity: body mass index (BMI) ≥25 kg/m^2^; (2) diagnosis of T2D and (3) presence of at least two of the following metabolic risk abnormalities: waist circumference ≥102/88 cm for men/women; blood pressure ≥130/85 mm Hg or specific drug treatment; plasma triglycerides≥150 mg/dL (1.70 mmol/L) or specific drug treatment; plasma high-density lipoprotein-cholesterol (HDL-C)<40 mg/dL (<1.0 mmol/L) for men and<50 mg/dL (<1.3 mmol/L) for women or specific drug treatment; pre-diabetes (fasting blood glucose: 5.6–6.9 mmol/L or HbA1c: 39–47 mmol/mol) and plasma high-sensitivity C reactive protein (CRP) level>2 mg/L. The diagnosis of MAFLD was conducted according to the new Delphi consensus.^1^ Participants were diagnosed with MASLD if they exhibited hepatic steatosis defined by PDFF≥5% and met at least one of the five cardiometabolic risk factors: (1) BMI≥25 kg/m^2^ or waist circumference≥102/88 cm for men/women; (2) fasting blood glucose≥5.6 mmol/L, HbA1c≥39 mmol/L or T2D and specific T2D treatment; (3) blood pressure≥130/85 mm Hg or specific drug treatment; (4) plasma triglycerides≥150 mg/dL (1.70 mmol/L) or specific drug treatment and (5) plasma HDL-C<40 mg/dL (<1.0 mmol/L) for men and <50 mg/dL (<1.3 mmol/L) for women or specific drug treatment.

### Accelerometry-measured physical activity

During 2013–2015, 236 519 UKB participants were invited to join the accelerometer substudy. Among them, 103 691 participants provided data collected using the Axivity AX3 triaxial wrist-worn triaxial accelerometer on their dominant hands, which captured their activity data over 7 days at a frequency of 100 Hz.^33^ Participants without good calibration and enough wear time were excluded from the study. Minutes per week of LPA, moderate-intensity physical activity (MPA) and vigorous-intensity physical activity (VPA) were defined as the time spent in 30–125 milligravities (mg), > 125–400 mg and >400 mg intensity activity, respectively,^27 34^ which were consistent with those used in previous studies involving UKB accelerometer data.

### Outcomes ascertainment

The primary outcomes of interest in our study were liver cirrhosis, cancer, CVD and death. Information on the occurrence and date of liver cirrhosis and CVD was obtained from hospital inpatient data. Information on the date and cause of death was obtained from the death registry. Information about cancer was obtained from the cancer register. The follow-up time began at the completion of accelerometry wearing and ended at the occurrence of the outcome or the end of follow-up (31 October 2022), whichever came first.

### Covariates ascertainment

We finally selected covariates, including age, sex, ethnicity, education level, the Townsend deprivation index (TDI), smoking status, alcohol intake frequency, diet scores, sleep scores, self-rated health status, BMI, waist circumference and other biochemical markers: triglycerides, HbA1c, HDL-C, blood glucose, blood pressure, high-sensitivity CRP level and Gamma-glutamyl transferase. Diet scores were generated based on the participant’s consumption frequency of fruits, vegetables, fish, processed meat and unprocessed red meat (0–5, a higher score indicates a better diet quality).^35^ Sleep scores were calculated based on the five sleep factors: chronotype, sleep duration, insomnia, snoring and excessive daytime sleepiness (0–5, a higher score indicates a better sleep quality).^36^ More details about diet and sleep scores are presented in online supplemental methods.

### Statistical analyses

Baseline characteristics of the participants were described as means and SDs for continuous variables and numbers (percentages) for categorical variables. The study evaluated dose–response associations of physical activity durations with liver cirrhosis incidence and all-cause mortality using restricted cubic splines fitted in the Cox proportional hazards models. The reference values were set at the first percentile of the physical activity duration distribution. After the comparison of AIC values for the optimal number of knots, five (for death: 0.05, 0.275, 0.50, 0.725 and 0.95 quantiles of the physical activity duration distribution) and three knots (for liver cirrhosis: 0.1, 0.5 and 0.9 quantiles of the physical activity distribution) were finally selected. Potential non-linearity was tested by Wald tests. Then, the physical activity duration was categorised into four levels considering the physical activity duration distributions, sample size and WHO recommendations (for MPA: 150 min/week and for VPA: 75 min/week). The physical activity durations were included in the Cox proportional hazard models as categorical variables to estimate HRs and 95% CIs. Linear trends were examined by entering the median value of each category of physical activity duration into the models. Three various variables-adjusted models were constructed. Model 1 was adjusted for age, sex and ethnicity. Model 2 was further adjusted for education level, TDI, smoking status, alcohol intake frequency, diet scores, sleep scores and self-rated health status. Model 3 was the primary model adjusted for all selected covariates. Considering the limited number of liver cirrhosis events, only model 1 and model 2 were used in the cirrhosis analyses. The proportional hazard assumption of the Cox models was examined by Schoenfield residuals and no violations were observed (online supplemental table 1). Additionally, a risk matrix was presented to investigate the associations of different physical activity duration combinations by physical activity intensity with all-cause mortality. The reference group was assigned to the combination of the lowest levels for physical activity duration. For cancer and CVD incidence, we found that the proportional assumption of Cox models was severely violated. Then, accelerated failure time models were constructed to evaluate the association between physical activity duration and these two outcomes, providing alternative estimates of survival. The physical activity duration of three intensities was included in the accelerated failure time models as continuous and categorical variables, respectively. All covariates were included in the accelerated failure time models. Stratified analyses were conducted according to age (<60 and ≥60), sex (female and male), smoking status (never and ever) and alcohol intake frequency (never and ever). For all-cause mortality, interaction terms were tested by Wald tests to examine whether associations varied by these factors. For cancer and CVD incidence, interaction terms were also added to the accelerated time models. Sensitivity analyses were conducted by the following two methods. First, we excluded participants whose outcome of interest came within the first 2 years of the follow-up to avoid potential risks of reverse causation. Second, participants with poor self-rated health status were excluded since they were less likely to do physical activities and faced a higher risk of adverse disease outcomes and mortality. Third, the model was adjusted for the medication use to further avoid confounding. Besides, we excluded individuals who were diagnosed with alcohol or viral hepatitis before baseline as two of the most common causes of cirrhosis within the UK to validate the findings. All the analyses were carried out using R software (V.4.4.2). The statistical significance was set as p<0.05 (two-sided test).

## Results

### Population characteristics

As shown in online supplemental figure 1, of the 502 366 UKB participants, 188 192 were diagnosed with MASLD at baseline. Of these 188 192 MASLD participants, accelerometer-measured physical activity data were available for 35 097 participants. By further excluding 2416 participants without calibration and enough wear time, 32 681 participants were left. Finally, 32 681, 21 919, 28 068 and 32 623 participants were available for all-cause mortality, CVD, cancers and liver cirrhosis analyses, respectively. Characteristics of these participants were shown in table 1.

**Table 1:** Baseline characteristics of participants with MASLD included in the analysis for four outcomes

	Death	CVD	Cancer	Liver cirrhosis
Total, n	32 681	21 919	28 068	32 623
Event, n	1883	6657	3312	151
Age, mean(SD)	63.04 (7.59)	61.84 (7.69)	62.52 (7.61）	63.04 (7.59)
Sex, male, n(**%**)	20 768 (63.55)	13 694 (62.43)	18 056 (64.32)	20 728 (63.54)
Ethnicity, white, n(**%**)	30 290 (92.68)	20 282 (92.47)	25 982 (92.56)	30 233 (92.67)
Education level, college or university, n(**%**)	12 137 (37.14)	8174 (39.73)	10 496 (37.39)	12 116 (37.14)
TDI, mean(SD)	−1.61 (2.88)	−1.66 (2.85)	−1.58 (2.90)	−1.61 (2.88)
Smoking status, n(**%**)				
Never	16 342 (50.00)	11 616 (52.96)	14 248 (50.76)	16 315 (50.01)
Previous	13 571 (41.53)	8449 (38.52)	11 423 (40.69)	13 548 (41.53)
Current	2686 (8.22)	1820 (8.30)	2329 (8.30)	2678 (8.21)
Prefer not to answer	82 (0.25)	49 (0.22)	71 (0.25)	82 (0.25)
Alcohol intake frequency, n(**%**)				
Daily or almost daily	7558 (23.13)	4980 (22.70)	6481 (23.09)	7546 (23.13)
Three or four times a week	8104 (24.80)	5622 (25.63)	6938 (24.72)	8095 (24.81)
One time or two times a week	8178 (25.02)	5650 (25.76)	7060 (25.15)	8171 (25.05)
One to three times a month	3643 (11.15)	2467 (11.25)	3150 (11.22)	3637 (11.15)
Special occasions only	3296 (10.09)	2070 (9.44)	2798 (9.97)	3287 (10.08)
Never	1890 (5.78)	1137 (5.18)	1633 (5.82)	1875 (5.75)
Prefer not to answer	12 (0.04)	8 (0.04)	11 (0.04)	12 (0.04)
Diet score, mean(SD)	2.65 (1.00)	2.64 (1.00)	2.64 (1.00）	2.65 (1.00)
Sleep score, mean(SD)	3.07 (1.00)	3.10 (0.97)	3.08 (0.99）	3.07 (1.00)
Self-rated health status, n(**%**)				
Excellent	4228 (12.94)	3403 (15.51)	3716 (13.24)	4224 (12.95)
Good	18 877 (58.07)	13 467 (61.40)	16 395 (58.41)	18 957 (58.11)
Fair	7876 (24.10)	4424 (20.17)	6628 (23.61)	7851 (24.07)
Poor	1500 (4.59)	587 (2.68)	1254 (4.47)	1491 (4.57)
Do not know	96 (0.29)	49 (0.22)	74 (0.26)	96 (0.29)
Prefer not to answer	4 (0.01)	4 (0.02)	4 (0.01)	4 (0.01)
BMI, mean(SD)	31.02 (4.41)	30.72 (4.22)	31.03 (4.41)	31.02 (4.41)
Waist circumference, mean(SD)	102.06 (9.89)	101.15 (9.47)	102.11 (9.92)	102.06 (9.89)
Plasma triglycerides, mean(SD)	2.35 (1.16)	2.36 (1.15)	2.35 (1.17)	2.35 (1.16)
HbA1c, mean(SD)	37.06 (7.17)	36.38 (6.39)	37.00 (7.23)	37.06 (7.16)
HDL-C, mean(SD)	1.29 (0.30)	1.30 (0.31)	1.29 (0.30)	1.29 (0.30)
Blood glucose, mean(SD)	5.28 (1.37)	5.19 (1.22)	5.27 (1.38)	5.28 (1.37)
Diastolic blood pressure, mean(SD)	88.28 (9.91)	88.30 (9.64)	88.37 (9.92)	88.28 (9.90)
Systolic blood pressure, mean(SD)	148.93 (18.56)	148.02 (18.23)	148.77 (18.50)	148.93 (18.56)
High-sensitive CRP, mean(SD)	3.33 (4.66)	3.18 (4.42)	3.29 (4.61）	3.33 (4.66)
GGT, mean(SD)	51.19 (50.91)	49.59 (47.40)	51.15 (50.39）	50.94 (49.84)

BMI, body mass index; CRP, C reactive protein; CVD, cardiovascular disease; GGT, Gamma-glutamyl transferase; HLD-C, high-density lipoprotein-cholesterol; MASLD, metabolic dysfunction-associated steatotic liver disease; TDI, Townsend deprivation index.

### Association of physical activity with all-cause mortality

**Figure 1:**
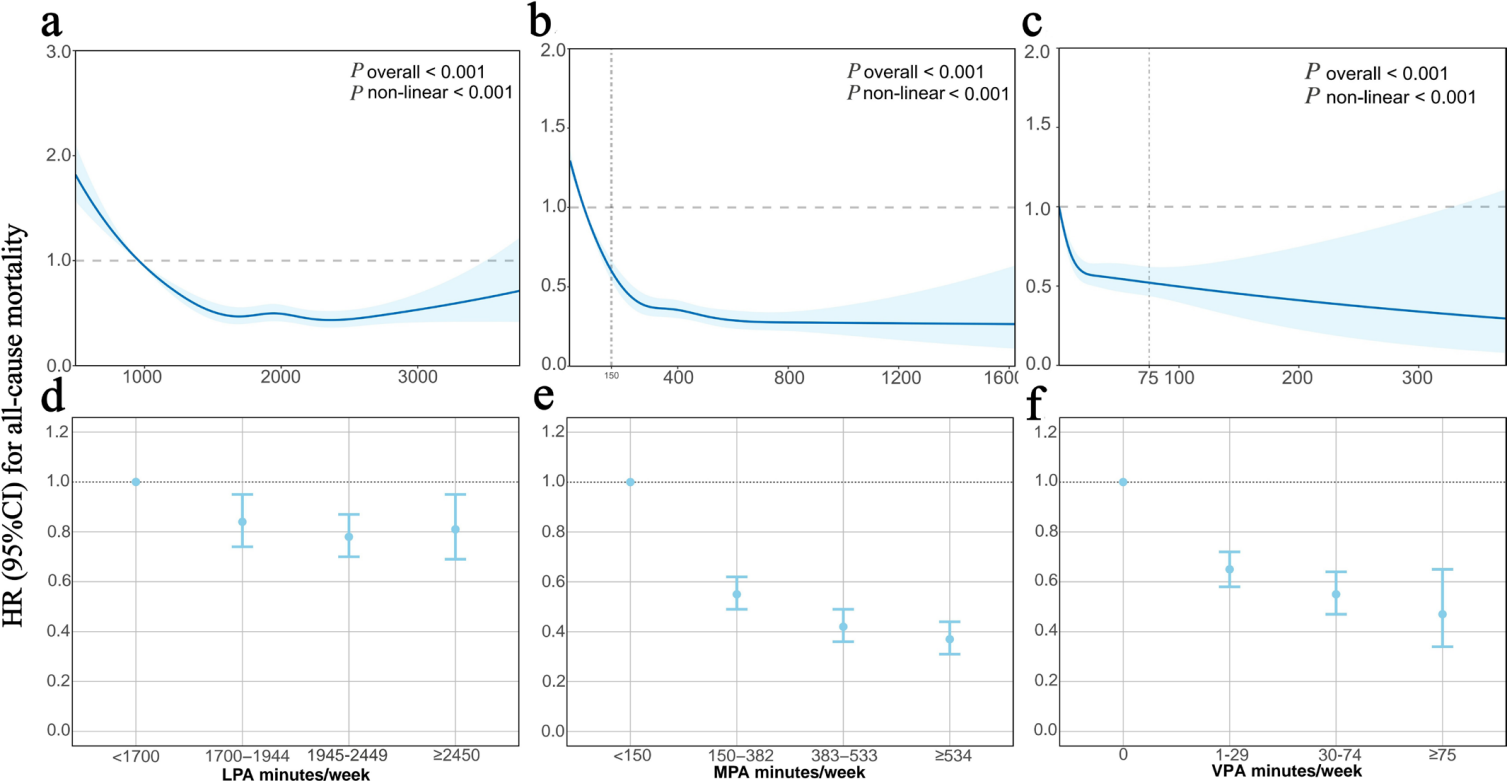
Associations of PA duration and all-cause mortality. HRs were calculated in Cox proportional hazards models adjusted for age, sex, ethnicity, education level, TDI, smoking status, alcohol intake frequency, diet scores, sleep scores, self-rated health status, BMI, waist circumference, triglycerides, HbA1c, HDL-C, blood glucose, blood pressure, high-sensitivity CRP level and GGT. (**a)–(c**) Dose–response associations of PA duration with all-cause mortality. Bold lines represent HRs, while shaded areas indicate 95% CI. Vertical grey dashed lines at 150 min/week of MPA and 75 min/week of VPA represent the WHO recommended durations for MPA and VPA. (d)–(f) HR (95% CI) of four classifications with PA duration as categorical variables. BMI, body mass index; CRP, C reactive protein; GGT, Gamma-glutamyl transferase; HDL-C, high-density lipoprotein-cholesterol; LPA, light-intensity physical activity; MPA, moderate-intensity physical activity; TDI, Townsend deprivation index; VPA, vigorous-intensity physical activity.

During a median follow-up of 7.9 years, 1883 death cases were recorded. The dose–response association of LPA with all-cause mortality was U-shaped, with an inflection point at about 2359 min/week (*p* value for non-linearity<0.001, figure 1A). By categorising LPA into four levels, the category of 1945–2449 min/week achieved with the lowest risk of all-cause mortality (HR=0.78, 95% CI 0.70 to 0.87) (table 2). For MPA and VPA, their dose–response associations with all-cause mortality were L-shaped (*p* values for non-linearity<0.001, figure 1B, C). As shown in table 2, categories of MPA≥534 min/week and VPA≥75 min/week achieved the lowest risk of all-cause mortality (HR=0.37, 95% CI 0.31 to 0.44 for MPA and HR=0.47, 95% CI 0.34 to 0.65 for VPA, respectively).

**Table 2:** HRs (95% CI) for all-cause mortality by PA intensity

Exposures	HR (95% CI)		
	Model 1	Model 2	Model 3
LPA (min/week)			
<1700	1(ref)	1(ref)	1(ref)
1700–1944	0.75 (0.66 to 0.85)	0.81 (0.71 to 0.92)	0.84 (0.74 to 0.95)
1945–2449	0.67 (0.60 to 0.75)	0.74 (0.66 to 0.83)	0.78 (0.70 to 0.87)
≥2450	0.68 (0.58 to 0.80)	0.76 (0.64 to 0.89)	0.81 (0.69 to 0.95)
*p* for trend	<0.001	<0.001	<0.001
MPA (min/week)			
<150	1(ref)	1(ref)	1(ref)
150–382	0.43 (0.38 to 0.49)	0.51 (0.45 to 0.57)	0.55 (0.49 to 0.62)
383–533	0.30 (0.26 to 0.35)	0.37 (0.32 to 0.44)	0.42 (0.36 to 0.49)
≥534	0.25 (0.22 to 0.30)	0.32 (0.27 to 0.38)	0.37 (0.31 to 0.44)
*p* for trend	<0.001	<0.001	<0.001
VPA (min/week)			
0	1(ref)	1(ref)	1(ref)
1–29	0.55 (0.49 to 0.60)	0.61 (0.55 to 0.67)	0.65 (0.58 to 0.72)
30–74	0.41 (0.35 to 0.47)	0.49 (0.43 to 0.57)	0.55 (0.47 to 0.64)
≥75	0.32 (0.23 to 0.44)	0.41 (0.29 to 0.56)	0.47 (0.34 to 0.65)
*p* for trend	<0.001	<0.001	<0.001

HRs were calculated in Cox proportional hazards models: model 1, adjusted for age, sex and ethnicity; Model 2, adjusted for age, sex, ethnicity, education level, TDI, smoking status, alcohol intake frequency, diet scores, sleep scores and self-rated health status; Model 3, adjusted for age, sex, ethnicity, education level, TDI, smoking status, alcohol intake frequency, diet scores, sleep scores, self-rated health status, BMI, waist circumstance, triglycerides, HbA1c, HDL-C, blood glucose, blood pressure, high-sensitivity CRP level and GGT.

BMI, body mass index; CRP, C reactive protein; GGT, Gamma-glutamyl transferase; HLD-C, high-density lipoprotein-cholesterol; LPA, light-intensity physical activity; MPA, moderate-intensity physical activity; PA, physical activity; TDI, Townsend deprivation index; VPA, vigorous-intensity physical activity.

**Figure 2:**
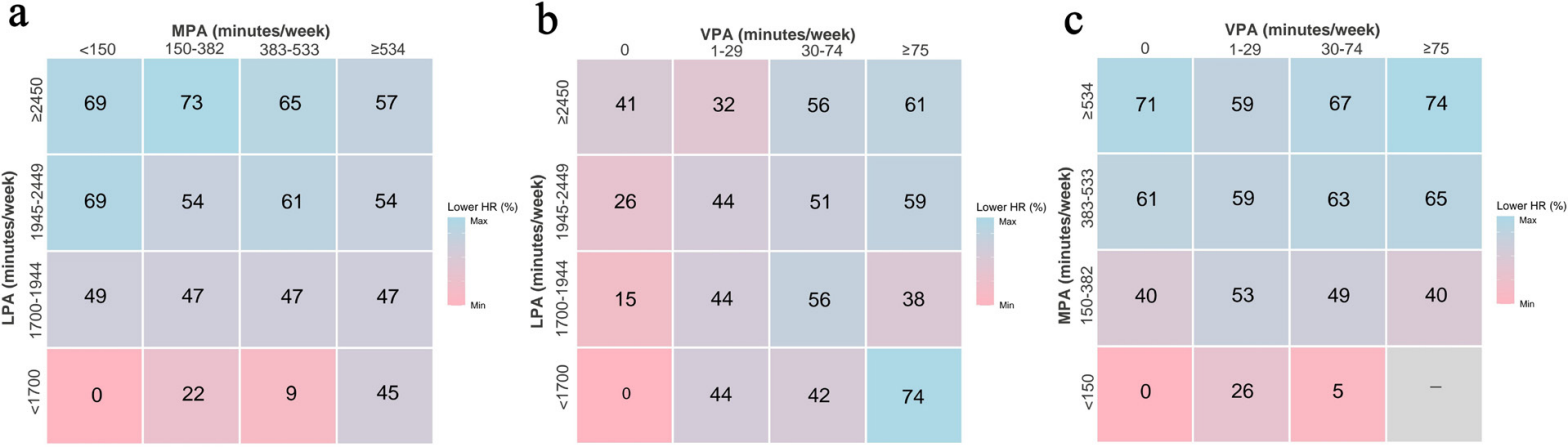
Risk matrix for the joint association between different combinations by intensity of PA duration and all-cause mortality. (a) The joint associations of LPA and MPA with all-cause mortality. (b) The joint associations of LPA and VPA with all-cause mortality. (c) The joint associations of MPA and VPA with all-cause mortality. HRs were calculated in Cox proportional hazards models: model 1, adjusted for age, sex and ethnicity; model 2, adjusted for age, sex, ethnicity, education level, TDI, smoking status, alcohol intake frequency, diet scores, sleep scores and self-rated health status; model 3, adjusted for age, sex, ethnicity, education level, TDI, smoking status, alcohol intake frequency, diet scores, sleep scores, self-rated health status, BMI, waist circumference, triglycerides, HbA1c, HDL-C, blood glucose, blood pressure, high-sensitivity CRP level and GGT. The numbers presented are the associated reduction in hazard (percentage) compared with the least active group, with a pinker colour indicating a lower risk reduction and a bluer colour representing a higher risk reduction. There were not enough participants to estimate HRs in the grey cell. BMI, body mass index; CRP, C reactive protein; GGT, Gamma-glutamyl transferase; HDL-C, high-density lipoprotein-cholesterol; LPA, light-intensity physical activity; MPA, moderate-intensity physical activity; PA, physical activity; TDI, Townsend deprivation index; VPA, vigorous-intensity physical activity.

We also evaluated the joint associations of different physical activities with all-cause mortality. As shown in figure 2 and online supplemental tables 2–4, combinations of 150–382 min/week of MPA and ≥2450 min/week of LPA (HR = 0.27; 95% CI 0.18 to 0.39), ≥75 min/week of VPA and<1700 min/week of LPA (HR = 0.26; 95% CI 0.11 to 0.62) and ≥75 min/week of VPA and≥534 min/week of MPA (HR = 0.26; 95% CI 0.16 to 0.40) would benefit most from physical activity.

### Comparison between MASLD individuals and non-MASLD individuals

**Figure 3:**
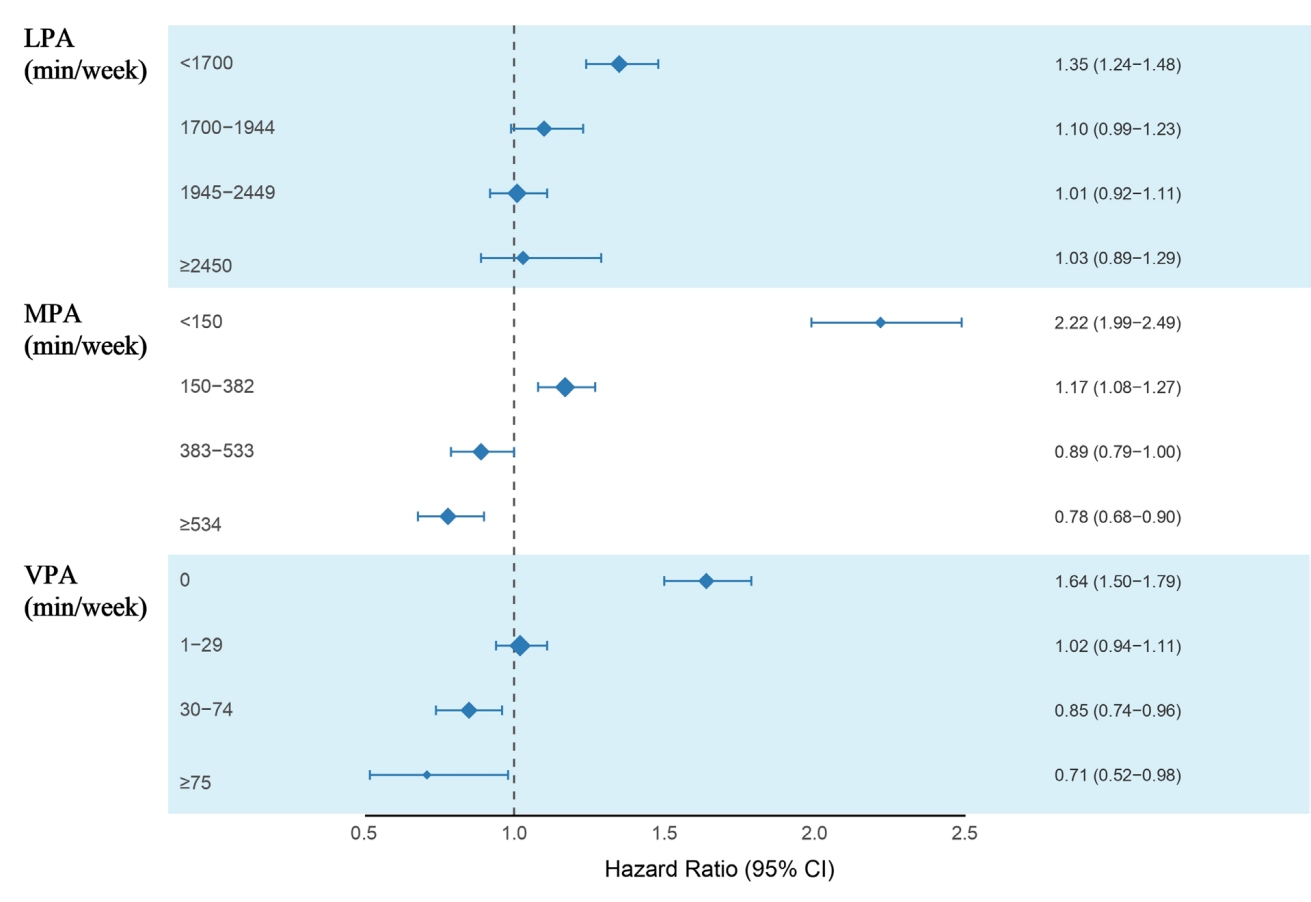
Mortality risk of MASLD individuals with different PA durations compared with the non-MASLD individuals. HRs were calculated in Cox proportional hazards models, with non-MASLD individuals as the reference (N=63 984), adjusted for age, sex, ethnicity, education level, TDI, smoking status, alcohol intake frequency, diet scores, sleep scores and self-rated health status. LPA, light-intensity physical activity; MASLD, metabolic-associated steatotic liver disease; MPA, moderate-intensity physical activity; PA, physical activity; TDI, Townsend deprivation index; VPA, vigorous-intensity physical activity.

In addition, using non-MASLD individuals from participants with accelerometer data (n=63 984) as controls, we examined the duration of physical activity required for MASLD individuals to achieve a mortality risk comparable with that of the MASLD-free population. As shown in figure 3, LPA durations of 1945–2449 minu/week reduced mortality risk in MALSD individuals to levels similar to those in the control cohort. A substantially lower threshold was observed for MPA, with 383 min/week sufficient to attenuate excess mortality risk to that of the control group. Notably, even minimal engagement in VPA conferred an equivalent reduction in mortality risk.

### Association of physical activity with liver cirrhosis incidence

**Figure 4:**
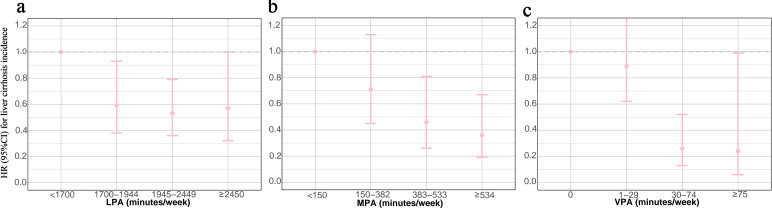
Associations of PA duration and liver cirrhosis incidence. HRs were calculated in Cox proportional hazards models adjusted for age, sex, ethnicity, education level, TDI, smoking status, alcohol intake frequency, diet scores, sleep scores and self-rated health status. (a)–(c) HR (95% CI) of four classifications with PA duration as categorical variables. LPA, light-intensity physical activity; MPA, moderate-intensity physical activity; PA, physical activity; TDI, Townsend deprivation index; VPA, vigorous-intensity physical activity.

During a median follow-up of 7.9 years, 151 liver cirrhosis cases were recorded. Dose–response relationships between physical activity and liver cirrhosis were linear for LPA and MPA, but not the case for VPA (online supplemental figure 2). When we categorised physical activities into four levels, the risk of liver cirrhosis generally decreased as the duration of physical activities increased, irrespective of physical activity intensity (all *p* values of trend test<0.001, table 3 and figure 4). For example, compared with the lowest level of MPA (<150 min/week), the HR of liver cirrhosis for 150–382 min/week, 383–533 min/week and ≥534 min/week was 0.71 (0.45–1.13), 0.46 (0.26–0.81) and 0.36 (0.19–0.67), respectively.

**Table 3:** HRs (95% CI) for liver cirrhosis incidence by PA intensity

Exposures	HR (95% CI)	
	Model 1	Model 2
LPA (min/week)		
<1700	1(ref)	1(ref)
1700–1944	0.52 (0.33 to 0.81)	0.59 (0.38 to 0.93)
1945–2449	0.44 (0.30 to 0.66)	0.53 (0.36 to 0.79)
≥2450	0.48 (0.27 to 0.84)	0.57 (0.32 to 1.00)
*p* for trend	<0.001	<0.001
MPA (min/week)		
<150	1(ref)	1(ref)
150–382	0.52 (0.33 to 0.82)	0.71 (0.45 to 1.13)
383–533	0.30 (0.17 to 0.51)	0.46 (0.26 to 0.81)
≥534	0.22 (0.12 to 0.41)	0.36 (0.19 to 0.67)
*p* for trend	<0.001	<0.001
VPA (min/week)		
0	1(ref)	1(ref)
1–29	0.74 (0.52 to 1.06)	0.89 (0.62 to 1.28)
30–74	0.19 (0.09 to 0.36)	0.26 (0.13 to 0.52)
≥75	0.15 (0.04 to 0.64)	0.24 (0.06 to 0.99)
*p* for trend	<0.001	<0.001

HRs were calculated in Cox proportional hazards models: model 1, adjusted for age, sex and ethnicity; Model 2, adjusted for age, sex, ethnicity, education level, TDI, smoking status, alcohol intake frequency, diet scores, sleep scores and self-rated health status.

LPA, light-intensity physical activity; MPA, moderate-intensity physical activity; PA, physical activity; TDI, Townsend deprivation index; VPA, vigorous-intensity physical activity.

### Association of physical activity with cancers and CVD

During a median follow-up of 7.8 and 7.5 years, 3312 cancer and 6657 CVD cases were documented. As the proportional hazard assumptions were violated in the analyses of physical activity with cancer and CVDs, accelerated failure time models were used. As shown in table 4, only VPA was significantly associated with a lower risk of cancer. Compared with the reference category (0 min/week), ≥75 min/week of VPA was estimated to have a 25% longer cancer-free survival (p=0.048). Both MPA and VPA were associated with lower risk of CVDs. As shown in table 4, compared with the reference group, both 383–533 min/week of MPA and MPA≥534 min/week were significantly associated with longer CVD-free survival (p=0.005 and p=0.011). For VPA, compared with the reference group, the estimates of 1–29 min/week, 30–74 min/week and≥75 min/week were 1.09 (p=0.006), 1.17 (p<0.001) and 1.20 (p=0.003), indicating 9%, 17% and 20% prolonged CVD-free survival, respectively.

**Table 4:** Accelerated failure time model demonstrating the association between PA and cancer/CVD survival by PA intensity

Exposures	Cancer (n=28 068)		CVD(n=21 919)	
	Estimate	P value	Estimate	P value
LPA (min/week)	1.00	0.408	1.00	0.380
LPA				
<1700	Ref	–	Ref	–
1700–1944	0.94	0.251	1.00	0.934
1945–2449	1.03	0.568	0.99	0.852
≥2450	1.02	0.757	0.95	0.162
MPA (min/week)	1.00	0.037	1.00	0.001
MPA				
<150	Ref	–	Ref	–
150–382	0.95	0.464	1.06	0.220
383–533	0.94	0.461	1.16	0.005
≥534	1.04	0.654	1.15	0.011
VPA (min/week)	1.00	0.055	1.00	0.001
VPA				
0	Ref	–	Ref	–
1–29	1.03	0.522	1.09	0.006
30–74	1.02	0.803	1.17	<0.001
≥75	1.25	0.048	1.20	0.003

Estimates were calculated in accelerated failure time models, which were all adjusted for age, sex, ethnicity, education level, TDI, smoking status, alcohol intake frequency, diet scores, sleep scores, self-rated health status, BMI, waist circumstance, triglycerides, HbA1c, HDL-C, blood glucose, blood pressure, high-sensitivity CRP level and GGT.

BMI, body mass index; CRP, C reactive protein; CVD, cardiovascular disease; GGT, Gamma-glutamyl transferase; HDL-C, high-density lipoprotein-cholesterol; LPA, light-intensity physical activity; MPA, moderate-intensity physical activity; PA, physical activity; TDI, Townsend deprivation index; VPA, vigorous-intensity physical activity.

### Stratified analyses and sensitivity analyses

For all-cause mortality, no statistically significant interactions were found between physical activity durations and age, sex, smoking status and alcohol intake (*p* for interaction>0.05, online supplemental tables 5-7). For liver cirrhosis, stratified analyses were not performed due to the limited number of cases. For cancers, no statistically significant interactions were found between MPA duration and age, sex, smoking status and alcohol intake (*p* for interaction>0.05, online supplemental table 8). For CVD, no statistically significant interactions were found between MPA duration and age, smoking status and alcohol intake (*p* for interaction>0.05, online supplemental table 9). However, females had longer CVD-free survival than males across all categories of VPA (p=0.0145, online supplemental table 10). Results of sensitivity analyses aligned with the main analyses for all-cause mortality, liver cirrhosis and cancers (online supplemental tables 11–14). But notably, excluding participants with CVD in the first 2 years revealed that LPA≥2450 min/week was associated with a higher risk of CVD (estimate=0.95, p<0.05).

## Discussion

In this prospective cohort study, a total of 32 681 adults with MASLD were included. We found that both VPA and≥383 min/week of MPA were significantly associated with a lower risk of CVDs. For liver cirrhosis, longer physical activity durations were associated with a reduced risk, regardless of intensity. For cancers, however, only VPA≥75 min/week showed a significant protective effect. Remarkably, physical activity was significantly associated with a lower risk of all-cause mortality, irrespective of activity intensity.

In our study, we observed a U-shaped dose–response association between accelerometer-derived LPA and all-cause mortality in patients with MASLD. This suggests that while LPA initially reduces mortality risk, its protective effects attenuate beyond a certain threshold. A similar pattern was noted in the analysis of cirrhosis, where prolonged LPA also exhibited a reduced protective effect. This attenuation phenomenon aligns with findings from a previous study investigating the association between physical activity and mortality risk in patients with T2D.^37^ We hypothesise that excessively prolonged durations of LPA may involve activities that inadvertently introduce risks for MASLD or T2D. Nevertheless, even short durations of LPA, when compared with complete inactivity, significantly reduce the risks of cirrhosis and mortality in patients with MASLD. For individuals who are inactive or unable to engage in MPA or VPA, initiating LPA represents a feasible and beneficial intervention. Both MPA and VPA exhibited L-shaped dose–response relationships with all-cause mortality. Specifically, we observed that a significant risk reduction was achieved with approximately 234 min/week of MPA, beyond which the benefits plateaued. In contrast, a notable decrease in all-cause mortality risk was associated with increasing VPA duration, up to a threshold of 20 min/week, after which the rate of risk reduction slowed. These findings indicate that exceeding 234 min/week of MPA results in diminishing returns regarding mortality risk reduction. In addition, a threshold of 20 min/week of VPA may be sufficient to confer significant health benefits, while also being more feasible for practical implementation. Also, compared with the control cohort, the duration of different intensities of physical activity suggests that, for MASLD individuals, engaging in VPA is the most effective way to reduce their mortality risk to levels comparable with the general non-MASLD population. We also evaluated the joint associations of different physical activities with all-cause mortality. Our findings suggest that combining multiple intensities of physical activity, rather than engaging in only one intensity, may provide greater benefits. Promoting a balanced mix of activities across different intensities could be an effective strategy to enhance the overall health and reduce mortality risk.

For cancer, only VPA durations of≥75 min/week were associated with a significantly beneficial effect, consistent with the WHO guidelines for VPA duration.^38^ For CVDs, MPA durations of at least 383 min/week were associated with beneficial effects, exceeding the WHO recommendation of 150 min/week for the general population. Therefore, for patients with MALSD attempting to prevent the onset or progression of CVD, more time needs to be invested for MPA than the general population. However, the benefits of VPA are achieved more quickly. Hence, patients with MASLD who are in good health status and willing to have VPA can experience quicker positive outcomes. These findings emphasise the importance of tailoring physical recommendations specifically for patients with MASLD.

Overall, studies examining the effects of physical activity in patients with MASLD remain limited. Only one study has reported that higher levels of leisure-time physical activity significantly reduce CVD risks among the MAFLD population.^35^ However, this study did not consider physical activity intensity and relied on self-reported data, making it unsuitable for direct comparison with our findings. Other studies, including physical activity and liver disease events, focus on the general population, rather than investigate the benefits of physical activity for MASLD individuals. To the best of our knowledge, our study is the first study to investigate the associations between physical activity and several adverse outcomes in MASLD populations using accelerometer-measured data. Accelerometers offer greater accuracy than self-report questionnaires by minimising recall bias, reducing intensity misclassification and improving the sensitivity in capturing LPA. The potential biological mechanisms underlying the association between physical activity and reduced risk of adverse outcomes, such as mortality, in patients with MASLD may include improved insulin sensitivity, better blood glucose control and reduced visceral fat accumulation—all of which contribute to a lower risk of liver cirrhosis and CVD.^39^ Additionally, given that MASLD represents a hepatic manifestation of metabolic disorders, physical activity-induced increases in cytokines, such as interleukin-6 and interleukin-10, may help alleviate chronic inflammation, thereby promoting the overall health.^40^

The strengths of this study were its large sample size and reliable data sources, which allowed for the accurate inclusion of patients with MASLD. Additionally, physical activity was objectively measured using accelerometers, enabling the capture of both leisure and non-leisure physical activity across various domains, thereby minimising recall bias and reporting bias. However, several limitations should be noted. First, the 7-day accelerometer measurement period may not fully reflect habitual physical activity patterns, and we cannot ascertain whether changes in physical activity habits occurred prior to the outcomes. Second, although wrist-worn accelerometers are more accurate than waist-worn accelerometers in differentiating activity intensities, they may not fully capture activities with minimal arm movement, such as cycling, which could be better quantified with ankle-worn accelerometers.^41^ However, these thresholds were selected out of subjectivity, and future research could benefit from more objective methods, such as machine-learning-based, data-driven approaches, to more accurately differentiate between activity intensities. Third, as an observational study, our findings are subject to the potential effects of unmeasured confounding, and the causal inferences cannot be drawn. Fourth, although there are more than 180 000 patients with MASLD diagnosed in the UKB, only approximately 30 000 have accelerometer-measured physical activity data, which introduces selection bias and may limit the generalisability of our findings. Future studies should validate the association between physical activity and adverse outcomes in patients with MASLD across diverse populations.

In conclusion, with accelerometer-derived physical activity data from the UKB, our study identified an association between physical activity duration and the risks of mortality and liver cirrhosis in patients with MASLD. Additionally, we identified a protective role of VPA at durations of ≥75 min/week in reducing cancer risk, as well as the preventive effects of both MPA and VPA on CVD incidence. These findings provide valuable reference for updating future physical activity guidelines, particularly by refining recommendations for MPA and VPA and incorporating guidance on LPA.

## Supplementary material

10.1136/bmjsem-2025-002702 online supplemental file 1

## Data Availability

Data are available on reasonable request.
