# Supplementary material for: Physical activity and the risk of cardiovascular disease, cirrhosis, cancer and mortality among individuals with MASLD: a prospective cohort study
Source: BMJ Open Sport Exerc Med. 2025 Oct 13;11(4):e002702. doi: 10.1136/bmjsem-2025-002702 (PMC12519692; doi:10.1136/bmjsem-2025-002702)
Supplement: online supplemental file 1 [file bmjsem-11-4-s001.docx]

**Supplementary methods**

***Multiple imputation***

We used multiple imputations by chained equations (MICE) to impute missing values for the following covariates: ethnicity, educational level, smoking status, alcohol intake frequency, self-rated health status, BMI, waist circumference, triglycerides, glycosylated hemoglobin (HbA1c), HDL-C, blood glucose, diastolic blood pressure, systolic blood pressure, high sensitivity C-reactive protein level and GGT. The imputation model included all of covariates as predictors.

***Diet score calculation***

(1) Consuming a minimum of 4 tablespoons of vegetables daily; (2) consuming at least 3 pieces of fruits daily; (3) consuming fish at least twice weekly; (4) consuming unprocessed red meat no more than twice weekly; (5) consuming processed meat no more than twice weekly. Each criterion earned 1 point and the diet score ranges from 0 to 5. A higher score indicates a more beneficial diet pattern.

***Sleep score calculation***

(1) sleep duration: 7-8 hours per day; (2) being a morning person; (3) never/rarely/sometimes insomnia; (4) not snoring; (5) experiencing never/rarely/sometimes excessive daytime sleepiness. The sleep score of each participant was calculated with self-reported questionnaires. The participant received 1 point for meeting one of the above standards and the sleep score ranges from 0 to 5. A higher score indicates a better sleep quality.

***Physical activity duration categorization***

The physical activity duration was categorized into four levels based on the quartile distribution of PA duration within our study population. For LPA, the boundaries were determined using rounded integers close to the empirical quartile values. For MPA and VPA, given that the first quartile or median values were below the WHO-recommended minimum thresholds (150 minutes/week for MPA and 75 minutes/week for VPA), we integrated these thresholds into the quartile-based categorization.

**Table M1. The distribution of accelerometer-derived physical activity duration among the MASLD individuals.**

|  | Minimum | First quartile | Median | Third quartile | Maximum |
| --- | --- | --- | --- | --- | --- |
| LPA (minutes) | 201.60 | 1663.20 | 1945.44 | 2447.84 | 4132.80 |
| MPA (minutes) | 0 | 262.08 | 383.04 | 534.24 | 2066.40 |
| VPA (minutes) | 0 | 10.08 | 10.08 | 30.24 | 695.52 |

**Supplementary Table 1. Results of Proportional Hazard tests in the primary models**

| Exposures | Outcomes | Global *p*-value |
| --- | --- | --- |
| LPA (minutes/week) | All-cause mortality | 0.157 |
| MPA (minutes/week) | All-cause mortality | 0.196 |
| VPA (minutes/week) | All-cause mortality | 0.248 |
| LPA (minutes/week) | Liver cirrhosis incidence | 0.349 |
| MPA (minutes/week) | Liver cirrhosis incidence | 0.330 |
| VPA (minutes/week) | Liver cirrhosis incidence | 0.354 |

For all-cause mortality, model 3 was used as the primary model. For liver cirrhosis, model 2 was used as the primary model. The proportional hazard assumption for Cox models was checked with Schoenfeld residuals.

**Supplementary Table 2. Joint associations of LPA and MPA with all-cause mortality for the risk matrix.**

| **Exposure**  **(minutes/week)** | | **Number of individuals** | **HR(95%CI)** |
| --- | --- | --- | --- |
| LPA | MPA | Total =32,681 | All-cause mortality |
| <1700 | <150 | 1725 | 1(ref) |
| <1700 | 150-382 | 363 | 0.78(0.58,1.04) |
| <1700 | 383-533 | 199 | 0.91(0.63,1.31) |
| <1700 | ≥534 | 22 | 0.55(0.14,2.22) |
| 1700-1944 | <150 | 5003 | 0.51(0.44,0.60) |
| 1700-1944 | 150-382 | 3372 | 0.53(0.45,0.63) |
| 1700-1944 | 383-533 | 4276 | 0.53(0.45,0.62) |
| 1700-1944 | ≥534 | 918 | 0.53(0.41,0.70) |
| 1945-2449 | <150 | 1563 | 0.31(0.23,0.42) |
| 1945-2449 | 150-382 | 1843 | 0.46(0.36,0.59) |
| 1945-2449 | 383-533 | 3762 | 0.39(0.32,0.48) |
| 1945-2449 | ≥534 | 1205 | 0.46(0.34,0.61) |
| ≥2450 | <150 | 868 | 0.31(0.20,0.48) |
| ≥2450 | 150-382 | 1358 | 0.27(0.18,0.39) |
| ≥2450 | 383-533 | 4003 | 0.35(0.28,0.44) |
| ≥2450 | ≥534 | 2201 | 0.43(0.33,0.56) |

Hazard ratios were calculated in Cox proportional hazards model after adjusting age, sex, ethnicity, education level, TDI, smoking status, alcohol intake frequency, diet scores, sleep scores, self-rated health status, BMI, waist circumstance and other biochemical markers: triglycerides, HbA1c, HDL-C, blood glucose, blood pressure, high-sensitivity CRP level and GGT.

HR: hazard ratio; CI: confidence interval; LPA: light-intensity physical activity; MPA: moderate-intensity physical activity; TDI: Townsend deprivation index; BMI: body mass index; HLD-C: high density lipoprotein cholesterol; CRP: C-reactive protein; GGT: Gamma-glutamyl transferase.

**Supplementary Table 3. Joint associations of LPA and VPA with all-cause mortality for the risk matrix.**

| **Exposure**  **(minutes/week)** | | **Number of individuals** | **HR(95%CI)** |
| --- | --- | --- | --- |
| LPA | VPA | Total =32,681 | All-cause mortality |
| <1700 | 0 | 3087 | 1(ref) |
| <1700 | 1-29 | 4322 | 0.56(0.48,0.66) |
| <1700 | 30-74 | 1401 | 0.58(0.44,0.76) |
| <1700 | ≥75 | 349 | 0.26(0.11,0.62) |
| 1700-1944 | 0 | 1421 | 0.85(0.70,1.03) |
| 1700-1944 | 1-29 | 3560 | 0.56(0.47,0.67) |
| 1700-1944 | 30-74 | 1612 | 0.44(0.33,0.59) |
| 1700-1944 | ≥75 | 343 | 0.62(0.34,1.13) |
| 1945-2449 | 0 | 1691 | 0.74(0.61,0.90) |
| 1945-2449 | 1-29 | 6229 | 0.56(0.48,0.65) |
| 1945-2449 | 30-74 | 3542 | 0.49(0.40,0.60) |
| 1945-2449 | ≥75 | 778 | 0.41(0.25,0.66) |
| ≥2450 | 0 | 485 | 0.59(0.40,0.86) |
| ≥2450 | 1-29 | 2140 | 0.68(0.56,0.84) |
| ≥2450 | 30-74 | 1421 | 0.44(0.32,0.61) |
| ≥2450 | ≥75 | 300 | 0.39(0.17,0.87) |

Hazard ratios were calculated in Cox proportional hazards model after adjusting age, sex, ethnicity, education level, TDI, smoking status, alcohol intake frequency, diet scores, sleep scores, self-rated health status, BMI, waist circumstance and other biochemical markers: triglycerides, HbA1c, HDL-C, blood glucose, blood pressure, high-sensitivity CRP level and GGT.

HR: hazard ratio; CI: confidence interval; LPA: light-intensity physical activity; VPA: vigorous-intensity physical activity; TDI: Townsend deprivation index; BMI: body mass index; HLD-C: high density lipoprotein cholesterol; CRP: C-reactive protein; GGT: Gamma-glutamyl transferase.

**Supplementary Table 4. Joint associations of MPA and VPA with all-cause mortality for the risk matrix.**

| **Exposure**  **(minutes/week)** | | **Number of individuals** | **HR(95%CI)** |
| --- | --- | --- | --- |
| MPA | VPA | Total =32,681 | All-cause mortality |
| <150 | 0 | 1902 | 1(ref) |
| <150 | 1-29 | 389 | 0.74(0.55,0.98) |
| <150 | 30-74 | 15 | 0.95(0.30,2.97) |
| <150 | ≥75 | 3 | * |
| 150-382 | 0 | 4099 | 0.60(0.52,0.70) |
| 150-382 | 1-29 | 8016 | 0.47(0.41,0.55) |
| 150-382 | 30-74 | 1276 | 0.51(0.39,0.66) |
| 150-382 | ≥75 | 178 | 0.60(0.30,1.22) |
| 383-533 | 0 | 549 | 0.39(0.26,0.59) |
| 383-533 | 1-29 | 4951 | 0.41(0.34,0.49) |
| 383-533 | 30-74 | 2498 | 0.37(0.29,0.48) |
| 383-533 | ≥75 | 375 | 0.35(0.19,0.67) |
| ≥534 | 0 | 134 | 0.29(0.11,0.79) |
| ≥534 | 1-29 | 2895 | 0.41(0.32,0.51) |
| ≥534 | 30-74 | 4187 | 0.33(0.26,0.41) |
| ≥534 | ≥75 | 1214 | 0.26(0.16,0.40) |

* There were not enough participants in this category for analysis.

Hazard ratios were calculated in Cox proportional hazards model after adjusting age, sex, ethnicity, education level, TDI, smoking status, alcohol intake frequency, diet scores, sleep scores, self-rated health status, BMI, waist circumstance and other biochemical markers: triglycerides, HbA1c, HDL-C, blood glucose, blood pressure, high-sensitivity CRP level and GGT.

HR: hazard ratio; CI: confidence interval; MPA: moderate-intensity physical activity; VPA: vigorous-intensity physical activity; TDI: Townsend deprivation index; BMI: body mass index; HLD-C: high density lipoprotein cholesterol; CRP: C-reactive protein; GGT: Gamma-glutamyl transferase.

**Supplementary Table 5. Associations between LPA duration and all-cause mortality in subgroups.**

| **Subgroups** | **LPA, minutes/week** | | | | ***p* for interaction** |
| --- | --- | --- | --- | --- | --- |
|  | <1700 | 1700-1944 | 1945-2449 | ≥2450 |  |
| **Age** |  |  |  |  | 0.9077 |
| <60 | 1(ref) | 0.65(0.42,1.00) | 0.64(0.44,0.92) | 0.70(0.44,1.12) |  |
| ≥60 | 1(ref) | 0.82(0.73,0.94) | 0.73(0.65,0.83) | 0.73(0.61,0.86) |  |
| **Sex** |  |  |  |  | 0.7440 |
| Female | 1(ref) | 0.88(0.76,1.02) | 0.77(0.68,0.88) | 0.88(0.72,1.06) |  |
| Male | 1(ref) | 0.71(0.55,0.92) | 0.77(0.62,0.96) | 0.66(0.49,0.89) |  |
| **Smoking status** |  |  |  |  | 0.3778 |
| Never | 1(ref) | 0.92(0.75,1.14) | 0.86(0.71,1.04) | 0.91(0.70,1.19) |  |
| Ever | 1(ref) | 0.80(0.68,0.93) | 0.73(0.64,0.84) | 0.75(0.61,0.92) |  |
| **Alcohol intake** |  |  |  |  | 0.0467 |
| Never | 1(ref) | 0.62(0.40,0.99) | 0.52(0.35,0.77) | 0.49(0.26,0.89) |  |
| Ever | 1(ref) | 0.86(0.76,0.98) | 0.80(0.71,0.90) | 0.84(0.71,1.00) |  |

Hazard ratios were calculated in Cox proportional hazards model after adjusting age, sex, ethnicity, education level, TDI, smoking status, alcohol intake frequency, diet scores, sleep scores, self-rated health status, BMI, waist circumstance and other biochemical markers: triglycerides, HbA1c, HDL-C, blood glucose, blood pressure, high-sensitivity CRP level and GGT.

HR: hazard ratio; CI: confidence interval; LPA: light-intensity physical activity; TDI: Townsend deprivation index; BMI: body mass index; HLD-C: high density lipoprotein cholesterol; CRP: C-reactive protein; GGT: Gamma-glutamyl transferase.

**Supplementary Table 6. Associations between MPA duration and all-cause mortality in subgroups.**

| **Subgroups** | **MPA, minutes/week** | | | | ***p* for interaction** |
| --- | --- | --- | --- | --- | --- |
|  | <150 | 150-382 | 383-533 | ≥534 |  |
| **Age** |  |  |  |  | 0.6954 |
| <60 | 1(ref) | 0.38(0.22,0.64) | 0.26(0.17,0.46) | 0.19(0.11,0.34) |  |
| ≥60 | 1(ref) | 0.48(0.42,0.54) | 0.37(0.28,0.40) | 0.28(0.23,0.34) |  |
| **Sex** |  |  |  |  | 0.2079 |
| Female | 1(ref) | 0.58(0.50,0.67) | 0.43(0.36,0.52) | 0.35(0.29,0.43) |  |
| Male | 1(ref) | 0.47(0.38,0.60) | 0.39(0.29,0.53) | 0.44(0.32,0.61) |  |
| **Smoking status** |  |  |  |  | 0.1171 |
| Never | 1(ref) | 0.52(0.41,0.64) | 0.45(0.35,0.59) | 0.43(0.32,0.57) |  |
| Ever | 1(ref) | 0.56(0.48,0.65) | 0.40(0.33,0.48) | 0.33(0.27,0.41) |  |
| **Alcohol intake** |  |  |  |  | 0.1018 |
| Never | 1(ref) | 0.39(0.27,0.57) | 0.31(0.18,0.53) | 0.28(0.15,0.54) |  |
| Ever | 1(ref) | 0.57(0.50,0.65) | 0.44(0.37,0.51) | 0.38(0.32,0.46) |  |

Hazard ratios were calculated in Cox proportional hazards model after adjusting age, sex, ethnicity, education level, TDI, smoking status, alcohol intake frequency, diet scores, sleep scores, self-rated health status, BMI, waist circumstance and other biochemical markers: triglycerides, HbA1c, HDL-C, blood glucose, blood pressure, high-sensitivity CRP level and GGT.

HR: hazard ratio; CI: confidence interval; MPA: moderate-intensity physical activity; TDI: Townsend deprivation index; BMI: body mass index; HLD-C: high density lipoprotein cholesterol; CRP: C-reactive protein; GGT: Gamma-glutamyl transferase.

**Supplementary Table 7. Associations between VPA duration and all-cause mortality in subgroups.**

| **Subgroups** | **VPA, minutes/week** | | | | ***p* for interaction** |
| --- | --- | --- | --- | --- | --- |
|  | 0 | 1-29 | 30-74 | ≥75 |  |
| **Age** |  |  |  |  | 0.6954 |
| <60 | 1(ref) | 0.61(0.42,0.90) | 0.38(0.24,0.60) | 0.23(0.10,0.53) |  |
| ≥60 | 1(ref) | 0.56(0.51,0.63) | 0.45(0.38,0.52) | 0.38(0.27,0.55) |  |
| **Sex** |  |  |  |  | 0.2079 |
| Female | 1(ref) | 0.64(0.57,0.72) | 0.52(0.44,0.62) | 0.46(0.32,0.66) |  |
| Male | 1(ref) | 0.66(0.54,0.80) | 0.68(0.50,0.93) | 0.49(0.20,1.19) |  |
| **Smoking status** |  |  |  |  | 0.1171 |
| Never | 1(ref) | 0.60(0.50,0.72) | 0.69(0.54,0.87) | 0.36(0.20,0.63) |  |
| Ever | 1(ref) | 0.66(0.58,0.75) | 0.46(0.38,0.56) | 0.54(0.36,0.81) |  |
| **Alcohol intake** |  |  |  |  | 0.1018 |
| Never | 1(ref) | 0.53(0.38,0.76) | 0.49(0.26,0.91) | 0.75(0.23,2.46) |  |
| Ever | 1(ref) | 0.66(0.59,0.73) | 0.55(0.47,0.65) | 0.46(0.32,0.64) |  |

Hazard ratios were calculated in Cox proportional hazards model after adjusting age, sex, ethnicity, education level, TDI, smoking status, alcohol intake frequency, diet scores, sleep scores, self-rated health status, BMI, waist circumstance and other biochemical markers: triglycerides, HbA1c, HDL-C, blood glucose, blood pressure, high-sensitivity CRP level and GGT.

HR: hazard ratio; CI: confidence interval; VPA: vigorous-intensity physical activity; TDI: Townsend deprivation index; BMI: body mass index; HLD-C: high density lipoprotein cholesterol; CRP: C-reactive protein; GGT: Gamma-glutamyl transferase.

**Supplementary Table 8. Association between VPA duration and cancer survival in subgroups.**

| **Subgroups** | **VPA, minutes/week, estimate (*p*)** | | ***p* for interaction** |
| --- | --- | --- | --- |
|  | 0 | ≥75 |  |
| **Age** |  |  | 0.2493 |
| <60 | 1(ref) | 1.26(0.235) |  |
| ≥60 | 1(ref) | 1.77(0.000) |  |
| **Sex** |  |  | 0.2301 |
| Female | 1(ref) | 1.22(0.456) |  |
| Male | 1(ref) | 1.24(0.087) |  |
| **Smoking status** |  |  | 0.6265 |
| Never | 1(ref) | 1.40(0.051) |  |
| Ever | 1(ref) | 1.14(0.383) |  |
| **Alcohol intake** |  |  | 0.2550 |
| Never | 1(ref) | 0.84(0.716) |  |
| Ever | 1(ref) | 1.29(0.034) |  |

Estimates were calculated in accelerated time failure model after adjusting age, sex, ethnicity, education level, TDI, smoking status, alcohol intake frequency, diet scores, sleep scores, self-rated health status, BMI, waist circumstance and other biochemical markers: triglycerides, HbA1c, HDL-C, blood glucose, blood pressure, high-sensitivity CRP level and GGT.

VPA: vigorous-intensity physical activity; TDI: Townsend deprivation index; BMI: body mass index; HLD-C: high density lipoprotein cholesterol; CRP: C-reactive protein; GGT: Gamma-glutamyl transferase.

**Supplementary Table 9. Association between MPA duration and CVD survival in subgroups.**

| **Subgroups** | **MPA, minutes/week, estimate (*p*)** | | | ***p* for interaction** |
| --- | --- | --- | --- | --- |
|  | <150 | 383-533 | ≥534 |  |
| **Age** |  |  |  | 0.7231 |
| <60 | 1(ref) | 1.32(0.067) | 1.25(0.145) |  |
| ≥60 | 1(ref) | 1.31(0.000) | 1.39(0.000) |  |
| **Sex** |  |  |  | 0.2523 |
| Female | 1(ref) | 1.17(0.055) | 1.15(0.096) |  |
| Male | 1(ref) | 1.16(0.034) | 1.15(0.046) |  |
| **Smoking status** |  |  |  | 0.8472 |
| Never | 1(ref) | 1.24(0.006) | 1.15(0.076) |  |
| Ever | 1(ref) | 1.12(0.123) | 1.17(0.029) |  |
| **Alcohol intake** |  |  |  | 0.0849 |
| Never | 1(ref) | 1.37(0.103) | 1.32(0.159) |  |
| Ever | 1(ref) | 1.14(0.014) | 1.13(0.027) |  |

Estimates were calculated in accelerated time failure model after adjusting age, sex, ethnicity, education level, TDI, smoking status, alcohol intake frequency, diet scores, sleep scores, self-rated health status, BMI, waist circumstance and other biochemical markers: triglycerides, HbA1c, HDL-C, blood glucose, blood pressure, high-sensitivity CRP level and GGT.

MPA: moderate-intensity physical activity; TDI: Townsend deprivation index; BMI: body mass index; HLD-C: high density lipoprotein cholesterol; CRP: C-reactive protein; GGT: Gamma-glutamyl transferase.

**Supplementary Table 10. Association between VPA duration and CVD survival in subgroups.**

| **Subgroups** | **VPA, minutes/week, estimate (*p*)** | | | | ***p* for interaction** |
| --- | --- | --- | --- | --- | --- |
|  | 0 | 1-29 | 30-74 | ≥75 |  |
| **Age** |  |  |  |  | 0.6947 |
| <60 | 1(ref) | 1.09(0.271) | 1.24(0.011) | 1.20(0.086) |  |
| ≥60 | 1(ref) | 1.17(0.000) | 1.30(0.000) | 1.51(0.000) |  |
| **Sex** |  |  |  |  | 0.0145 |
| Female | 1(ref) | 1.09(0.071) | 1.28(0.000) | 1.59(0.002) |  |
| Male | 1(ref) | 1.08(0.061) | 1.12(0.017) | 1.12(0.105) |  |
| **Smoking status** |  |  |  |  | 0.8682 |
| Never | 1(ref) | 1.09(0.054) | 1.10(0.065) | 1.22(0.028) |  |
| Ever | 1(ref) | 1.09(0.035) | 1.25(0.000) | 1.20(0.038) |  |
| **Alcohol intake** |  |  |  |  | 0.0506 |
| Never | 1(ref) | 1.18(0.149) | 1.36(0.054) | 0.88(0.570) |  |
| Ever | 1(ref) | 1.08(0.015) | 1.16(0.000) | 1.23(0.002) |  |

Estimates were calculated in accelerated time failure model after adjusting age, sex, ethnicity, education level, TDI, smoking status, alcohol intake frequency, diet scores, sleep scores, self-rated health status, BMI, waist circumstance and other biochemical markers: triglycerides, HbA1c, HDL-C, blood glucose, blood pressure, high-sensitivity CRP level and GGT.

VPA: vigorous-intensity physical activity; TDI: Townsend deprivation index; BMI: body mass index; HLD-C: high density lipoprotein cholesterol; CRP: C-reactive protein; GGT: Gamma-glutamyl transferase.

**Supplementary Table 11. Association of PA duration with all-cause mortality and liver cirrhosis incidence, excluding patients with within-2-year outcome or poor self-rated health.**

|  | **All-cause mortality** | | **Liver cirrhosis incidence** | |
| --- | --- | --- | --- | --- |
| **Total, n** | 32,426^A^ | 31,181^B^ | 32,598^A^ | 311,32^B^ |
| **LPA, minutes/week** |  |  |  |  |
| <1700 | 1(ref) | 1(ref) | 1(ref) | 1(ref) |
| 1700-1944 | 0.89(0.78,1.02) | 0.84(0.73,0.96) | 0.66(0.41,1.07) | 0.53(0.32,0.87) |
| 1945-2449 | 0.83(0.74,0.94) | 0.81(0.72,0.91) | 0.60(0.39,0.92) | 0.57(0.38,0.86) |
| ≥2450 | 0.84(0.71,1.00) | 0.85(0.72,1.01) | 0.56(0.30,1.06) | 0.51(0.27,0.97) |
| **MPA, minutes/week** |  |  |  |  |
| <150 | 1(ref) | 1(ref) | 1(ref) | 1(ref) |
| 150-382 | 0.57(0.50,0.65) | 0.56(0.49,0.64) | 0.83(0.49,1.41) | 0.58(0.35,0.96) |
| 383-533 | 0.46(0.39,0.54) | 0.44(0.37,0.52) | 0.50(0.26,0.95) | 0.41(0.22,0.75) |
| ≥534 | 0.40(0.34,0.49) | 0.40(0.33,0.48) | 0.40(0.20,0.81) | 0.32(0.16,0.62) |
| **VPA, minutes/week** |  |  |  |  |
| 0 | 1(ref) | 1(ref) | 1(ref) | 1(ref) |
| 1-29 | 0.67(0.60,0.75) | 0.66(0.59,0.73) | 1.00(0.67,1.50) | 0.93(0.62,1.38) |
| 30-74 | 0.58(0.50,0.68) | 0.56(0.48,0.65) | 0.24(0.11,0.53) | 0.18(0.08,0.41) |
| ≥75 | 0.47(0.33,0.67) | 0.51(0.36,0.71) | 0.30(0.07,1.27) | 0.26(0.06,0.19) |

Hazard ratios were calculated in Cox proportional hazards models. The model for all-cause mortality was adjusted for age, sex, ethnicity, education level, TDI, smoking status, alcohol intake frequency, diet scores, sleep scores, self-rated health status, BMI, waist circumstance, triglycerides, HbA1c, HDL-C, blood glucose, blood pressure, high-sensitivity CRP level and GGT. The model for liver cirrhosis incidence was adjusted for age, sex, ethnicity, education level, TDI, smoking status, alcohol intake frequency, diet scores, sleep scores and self-rated health status.

HR: hazard ratio; CI: confidence interval; LPA: light-intensity physical activity; MPA: moderate-intensity physical activity; VPA: vigorous-intensity physical activity; TDI: Townsend deprivation index; BMI: body mass index; HLD-C: high density lipoprotein cholesterol; CRP: C-reactive protein; GGT: Gamma-glutamyl transferase.

A: after excluding patients with within-2-year outcomes.

B: after excluding patients with poor self-rated health.

**Supplementary Table 12. Association of PA duration with cancer and CVD survival, excluding patients with within-2-year outcome or poor self-rated health.**

| **Exposures** | **Cancer** | | **CVD** | |
| --- | --- | --- | --- | --- |
|  | 27,047^A^ | 26,814^B^ | 20,068^A^ | 21,332^B^ |
| **LPA (minutes/week)** | 1.00(0.878) | 1.00(0.561) | 1.00(0.119) | 1.00(0.421) |
| **LPA** |  |  |  |  |
| <1700 | Ref | Ref | Ref | Ref |
| 1700-1944 | 0.94(0.092) | 0.94(0.307) | 0.98(0.473) | 0.99(0.878) |
| 1945-2449 | 0.98(0.593) | 1.03(0.588) | 0.98(0.303) | 0.99(0.775) |
| ≥2450 | 1.01(0.889) | 1.01(0.939) | 0.95(0.038) | 0.95(0.161) |
| **MPA (minutes/week)** | 1.00(0.209) | 1.00(0.058) | 1.00(0.032) | 1.00(0.000) |
| **MPA** |  |  |  |  |
| <150 | Ref | Ref | Ref | Ref |
| 150-382 | 0.93(0.202) | 0.93(0.393) | 0.97(0.320) | 1.08(0.142) |
| 383-533 | 0.90(0.079) | 0.93(0.420) | 1.01(0.716) | 1.18(0.002) |
| ≥534 | 0.98(0.719) | 1.02(0.866) | 1.00(0.927) | 1.16(0.006) |
| **VPA (minutes/week)** | 1.00(0.059) | 1.00(0.046) | 1.00(0.046) | 1.00(0.001) |
| **VPA** |  |  |  |  |
| **0** | Ref | Ref | Ref | Ref |
| **1-29** | 0.99(0.765) | 1.04(0.504) | 1.04(0.029) | 1.10(0.003) |
| **30-74** | 0.97(0.514) | 1.02(0.755) | 1.08(0.002) | 1.18(0.000) |
| **≥75** | 1.18(0.046) | 1.26(0.047) | 1.05(0.231) | 1.21(0.002) |

Estimates were calculated in accelerated time failure model after adjusting age, sex, ethnicity, education level, TDI, smoking status, alcohol intake frequency, diet scores, sleep scores, self-rated health status, BMI, waist circumstance and other biochemical markers: triglycerides, HbA1c, HDL-C, blood glucose, blood pressure, high-sensitivity CRP level and GGT.

LPA: light-intensity physical activity; MPA: moderate-intensity physical activity; VPA: vigorous-intensity physical activity; TDI: Townsend deprivation index; BMI: body mass index; HLD-C: high density lipoprotein cholesterol; CRP: C-reactive protein; GGT: Gamma-glutamyl transferase.

A: after excluding patients with within-2-year outcomes.

B: after excluding patients with poor self-rated health.

**Supplementary Table 13. Association of PA duration with all-cause mortality and liver cirrhosis incidence, adjusted further for medication use.**

|  | **All-cause mortality** | **Liver cirrhosis incidence** |
| --- | --- | --- |
| **Total, n** | 32677 | 32619 |
| **LPA, minutes/week** |  |  |
| <1700 | 1(ref) | 1(ref) |
| 1700-1944 | 0.84(0.74,0.95) | 0.59(0.38,0.93) |
| 1945-2449 | 0.78(0.70,0.87) | 0.54(0.36,0.80) |
| ≥2450 | 0.81(0.69,0.95) | 0.57(0.32,1.00) |
| **MPA, minutes/week** |  |  |
| <150 | 1(ref) | 1(ref) |
| 150-382 | 0.55 (0.49,0.62) | 0.71(0.45,1.13) |
| 383-533 | 0.42 (0.36,0.49) | 0.46(0.26,0.82) |
| ≥534 | 0.37 (0.31,0.44) | 0.36(0.19,0.68) |
| **VPA, minutes/week** |  |  |
| 0 | 1(ref) | 1(ref) |
| 1-29 | 0.65(0.58,0.72) | 0.90(0.62,1.28) |
| 30-74 | 0.55(0.47,0.64) | 0.27(0.13,0.52) |
| ≥75 | 0.47(0.34,0.65) | 0.24(0.06,1.02) |

Hazard ratios were calculated in Cox proportional hazards models. The model for all-cause mortality was adjusted for age, sex, ethnicity, education level, TDI, smoking status, alcohol intake frequency, diet scores, sleep scores, self-rated health status, BMI, waist circumstance, triglycerides, HbA1c, HDL-C, blood glucose, blood pressure, high-sensitivity CRP level, GGT and medication use. The model for liver cirrhosis incidence was adjusted for age, sex, ethnicity, education level, TDI, smoking status, alcohol intake frequency, diet scores, sleep scores, self-rated health status and medication use.

HR: hazard ratio; CI: confidence interval; LPA: light-intensity physical activity; MPA: moderate-intensity physical activity; VPA: vigorous-intensity physical activity; TDI: Townsend deprivation index; BMI: body mass index; HLD-C: high density lipoprotein cholesterol; CRP: C-reactive protein; GGT: Gamma-glutamyl transferase.

**Supplementary Table 14. Association of PA duration with cancer and CVD survival, adjusted further for medication use.**

|  | **Cancer** | **CVD** |
| --- | --- | --- |
| **Total, n** | 28064 | 21916 |
| **LPA, minutes/week** |  |  |
| <1700 | ref | ref |
| 1700-1944 | 0.94(0.251) | 0.99(0.872) |
| 1945-2449 | 1.03(0.559) | 0.99(0.836) |
| ≥2450 | 1.02(0.757) | 0.94(0.147) |
| **MPA, minutes/week** |  |  |
| <150 | ref | ref |
| 150-382 | 0.95(0.462) | 1.05(0.284) |
| 383-533 | 0.94(0.465) | 1.15(0.009) |
| ≥534 | 1.04(0.660) | 1.13(0.019) |
| **)VPA, minutes/week** |  |  |
| 0 | ref | ref |
| 1-29 | 1.03(0.518) | 1.08(0.009) |
| 30-74 | 1.02(0.816) | 1.16(0.000) |
| ≥75 | 1.25(0.049) | 1.18(0.007) |

Estimates were calculated in accelerated time failure model after adjusting age, sex, ethnicity, education level, TDI, smoking status, alcohol intake frequency, diet scores, sleep scores, self-rated health status, BMI, waist circumstance and other biochemical markers: triglycerides, HbA1c, HDL-C, blood glucose, blood pressure, high-sensitivity CRP level, GGT and medication use.

LPA: light-intensity physical activity; MPA: moderate-intensity physical activity; VPA: vigorous-intensity physical activity; TDI: Townsend deprivation index; BMI: body mass index; HLD-C: high density lipoprotein cholesterol; CRP: C-reactive protein; GGT: Gamma-glutamyl transferase.

**Supplementary Figure 1. Flowchart of participants inclusion.**

**
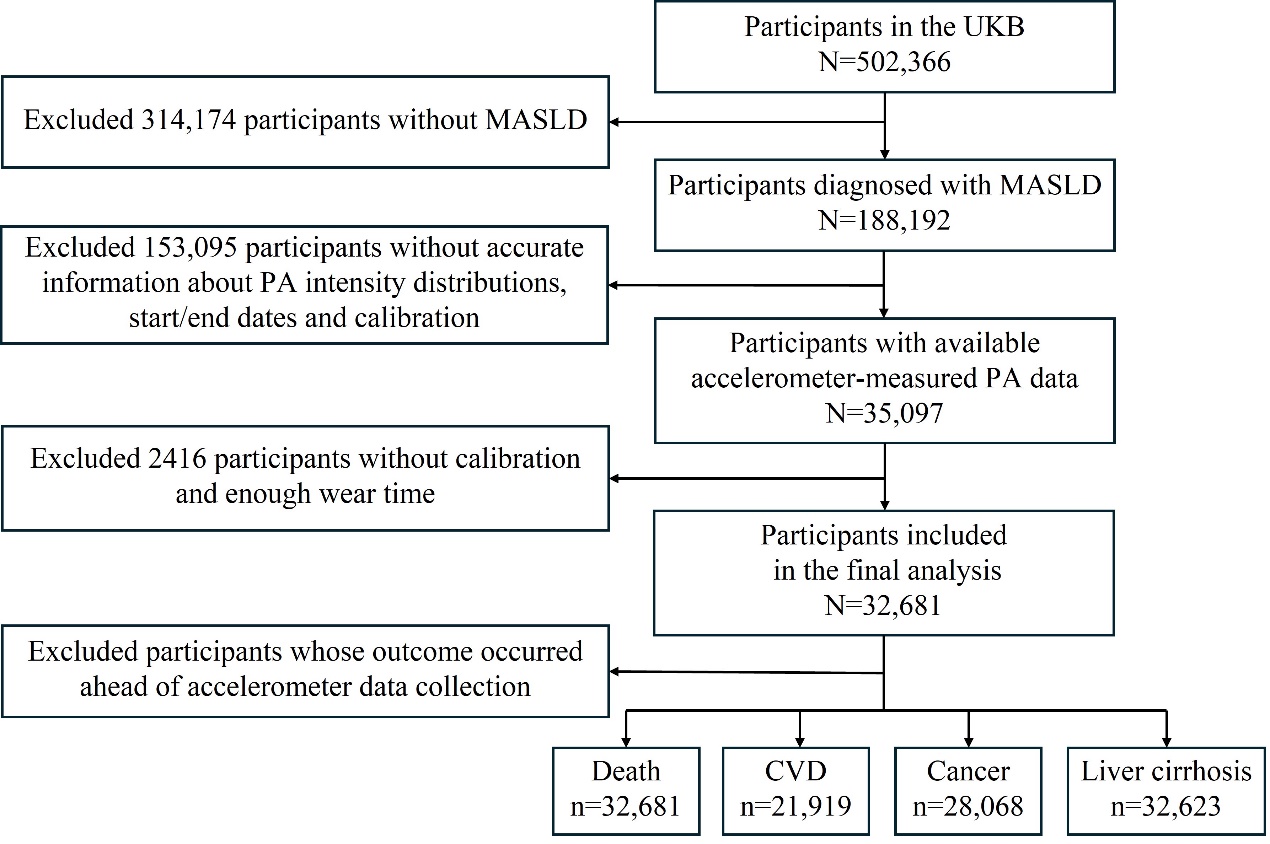
**

PA: physical activity; CVD: cardiovascular diseases.

**Supplementary Figure 2. Dose-response association between PA duration and liver cirrhosis incidence.**


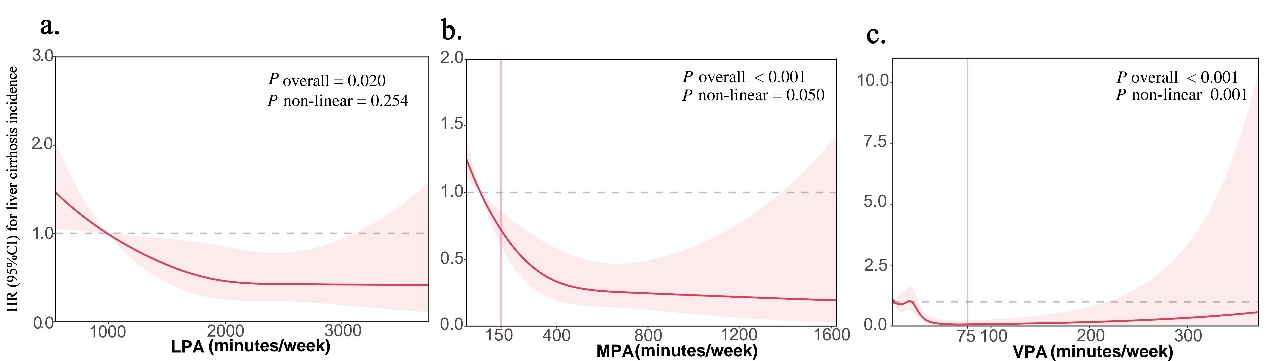


Hazard ratios were calculated in Cox proportional hazards model after adjusting age, sex, ethnicity, education level, TDI, smoking status, alcohol intake frequency, diet scores, sleep scores, and self-rated health status.

**a-c:** dose-response associations of PA duration with all-cause mortality. Bold lines represent HRs, while shaded areas indicate 95%CI. Bold lines represent HRs, while shaded areas indicate 95%CI. Vertical gray dashed lines at 150 minutes/week of MPA and 75 minutes/week of VPA represent the WHO recommended durations for MPA and VPA.

HR: hazard ratio; CI: confidence interval; LPA: light-intensity physical activity; MPA: moderate-intensity physical activity; VPA: vigorous-intensity physical activity; TDI: Townsend deprivation index.
